# Supplementary material for: A Critical View of the Application of the APEX Software (Aqueous Photochemistry of Environmentally-Occurring Xenobiotics) to Predict Photoreaction Kinetics in Surface Freshwaters
Source: Molecules. 2019 Dec 18;25(1):9. doi: 10.3390/molecules25010009 (PMC7017383; doi:10.3390/molecules25010009)
Supplement: Supplementary file 1 [file molecules-25-00009-s001.zip › 3.2.4_gcc-4.4.0.zip!/3.2.4_gcc-4.4.0/3.2.4_gcc-4.4.0/doc/octave/html/interpreter/Enabling-and-Disabling-Warnings.html]

Enabling and Disabling Warnings - Untitled


Previous: Issuing Warnings,
Up: Handling Warnings


---

#### 12.2.2 Enabling and Disabling Warnings

The `warning` function also allows you to control which warnings
are actually printed to the screen. If the `warning` function
is called with a string argument that is either `"on"` or `"off"`
all warnings will be enabled or disabled.

It is also possible to enable and disable individual warnings through
their string identifications. The following code will issue a warning

```
     warning ("non-negative-variable",
              "'a' must be non-negative.  Setting 'a' to zero.");
```

while the following won't issue a warning

```
     warning ("off", "non-negative-variable");
     warning ("non-negative-variable",
              "'a' must be non-negative.  Setting 'a' to zero.");
```

The functions distributed with Octave can issue one of the following
warnings.

`Octave:array-to-scalar`: If the `Octave:array-to-scalar` warning is enabled, Octave will warn when an implicit conversion from an array to a scalar value is attempted. By default, the `Octave:array-to-scalar` warning is disabled. `Octave:array-to-vector`: If the `Octave:array-to-vector` warning is enabled, Octave will warn when an implicit conversion from an array to a vector value is attempted. By default, the `Octave:array-to-vector` warning is disabled. `Octave:assign-as-truth-value`: If the `Octave:assign-as-truth-value` warning is enabled, a warning is issued for statements like ``` if (s = t) ... ``` since such statements are not common, and it is likely that the intent was to write ``` if (s == t) ... ``` instead. There are times when it is useful to write code that contains assignments within the condition of a `while` or `if` statement. For example, statements like ``` while (c = getc()) ... ``` are common in C programming. It is possible to avoid all warnings about such statements by disabling the `Octave:assign-as-truth-value` warning, but that may also let real errors like ``` if (x = 1) # intended to test (x == 1)! ... ``` slip by. In such cases, it is possible suppress errors for specific statements by writing them with an extra set of parentheses. For example, writing the previous example as ``` while ((c = getc())) ... ``` will prevent the warning from being printed for this statement, while allowing Octave to warn about other assignments used in conditional contexts. By default, the `Octave:assign-as-truth-value` warning is enabled. `Octave:associativity-change`: If the `Octave:associativity-change` warning is enabled, Octave will warn about possible changes in the meaning of some code due to changes in associativity for some operators. Associativity changes have typically been made for matlab compatibility. By default, the `Octave:associativity-change` warning is enabled. `Octave:divide-by-zero`: If the `Octave:divide-by-zero` warning is enabled, a warning is issued when Octave encounters a division by zero. By default, the `Octave:divide-by-zero` warning is enabled. `Octave:empty-list-elements`: If the `Octave:empty-list-elements` warning is enabled, a warning is issued when an empty matrix is found in a matrix list. For example, ``` a = [1, [], 3, [], 5] ``` By default, the `Octave:empty-list-elements` warning is enabled. `Octave:fortran-indexing`: If the `Octave:fortran-indexing` warning is enabled, a warning is printed for expressions which select elements of a two-dimensional matrix using a single index. By default, the `Octave:fortran-indexing` warning is disabled. `Octave:function-name-clash`: If the `Octave:function-name-clash` warning is enabled, a warning is issued when Octave finds that the name of a function defined in a function file differs from the name of the file. (If the names disagree, the name declared inside the file is ignored.) By default, the `Octave:function-name-clash` warning is enabled. `Octave:future-time-stamp`: If the `Octave:future-time-stamp` warning is enabled, Octave will print a warning if it finds a function file with a time stamp that is in the future. By default, the `Octave:future-time-stamp` warning is enabled. `Octave:imag-to-real`: If the `Octave:imag-to-real` warning is enabled, a warning is printed for implicit conversions of complex numbers to real numbers. By default, the `Octave:imag-to-real` warning is disabled. `Octave:matlab-incompatible`: Print warnings for Octave language features that may cause compatibility problems with matlab. `Octave:missing-semicolon`: If the `Octave:missing-semicolon` warning is enabled, Octave will warn when statements in function definitions don't end in semicolons. By default the `Octave:missing-semicolon` warning is disabled. `Octave:neg-dim-as-zero`: If the `Octave:neg-dim-as-zero` warning is enabled, print a warning for expressions like ``` eye (-1) ``` By default, the `Octave:neg-dim-as-zero` warning is disabled. `Octave:num-to-str`: If the `Octave:num-to-str` warning is enable, a warning is printed for implicit conversions of numbers to their ASCII character equivalents when strings are constructed using a mixture of strings and numbers in matrix notation. For example, ``` [ "f", 111, 111 ] => "foo" ``` elicits a warning if the `Octave:num-to-str` warning is enabled. By default, the `Octave:num-to-str` warning is enabled. `Octave:precedence-change`: If the `Octave:precedence-change` warning is enabled, Octave will warn about possible changes in the meaning of some code due to changes in precedence for some operators. Precedence changes have typically been made for matlab compatibility. By default, the `Octave:precedence-change` warning is enabled. `Octave:reload-forces-clear`: If several functions have been loaded from the same file, Octave must clear all the functions before any one of them can be reloaded. If the `Octave:reload-forces-clear` warning is enabled, Octave will warn you when this happens, and print a list of the additional functions that it is forced to clear. By default, the `Octave:reload-forces-clear` warning is enabled. `Octave:resize-on-range-error`: If the `Octave:resize-on-range-error` warning is enabled, print a warning when a matrix is resized by an indexed assignment with indices outside the current bounds. By default, the `Octave:resize-on-range-error` warning is disabled. `Octave:separator-insert`: Print warning if commas or semicolons might be inserted automatically in literal matrices. `Octave:single-quote-string`: Print warning if a single quote character is used to introduce a string constant. `Octave:str-to-num`: If the `Octave:str-to-num` warning is enabled, a warning is printed for implicit conversions of strings to their numeric ASCII equivalents. For example, ``` "abc" + 0 => 97 98 99 ``` elicits a warning if the `Octave:str-to-num` warning is enabled. By default, the `Octave:str-to-num` warning is disabled. `Octave:string-concat`: If the `Octave:string-concat` warning is enabled, print a warning when concatenating a mixture of double and single quoted strings. By default, the `Octave:string-concat` warning is disabled. `Octave:undefined-return-values`: If the `Octave:undefined-return-values` warning is disabled, print a warning if a function does not define all the values in the return list which are expected. By default, the `Octave:undefined-return-values` warning is enabled. `Octave:variable-switch-label`: If the `Octave:variable-switch-label` warning is enabled, Octave will print a warning if a switch label is not a constant or constant expression. By default, the `Octave:variable-switch-label` warning is disabled.
